# Hypogonadotropic Hypogonadism Before and After Transsphenoidal Surgery for Non‐Functioning Pituitary Adenoma

**DOI:** 10.1111/cen.70139

**Published:** 2026-04-06

**Authors:** Victor Hantelius, Gudmundur Johannsson, Sofie Jakobsson, Erik Thurin, Tobias Hallen, Thomas Skoglund, Oskar Ragnarsson

**Affiliations:** ^1^ Department of Internal Medicine and Clinical Nutrition, Institute of Medicine, Sahlgrenska Academy University of Gothenburg Gothenburg Sweden; ^2^ Department of Medicine Sahlgrenska University Hospital Gothenburg Sweden; ^3^ Endocrinology, Sahlgrenska University Hospital ENDO‐ERN (European Reference Network on Rare Endocrine Conditions) and University of Gothenburg Gothenburg Sweden; ^4^ University of Gothenburg Centre for Person‐Centred Care (GPCC) Gothenburg Sweden; ^5^ Institute of Health and Care Sciences, The Sahlgrenska Academy University of Gothenburg Gothenburg Sweden; ^6^ Department of Clinical Neuroscience, Institute of Neuroscience and Physiology, Sahlgrenska Academy University of Gothenburg Gothenburg Sweden; ^7^ Department of Radiology Sahlgrenska University Hospital Gothenburg Sweden; ^8^ Department of Neurosurgery Sahlgrenska University Hospital Gothenburg Sweden; ^9^ Wallenberg Centre for Molecular and Translational Medicine University of Gothenburg Gothenburg Sweden

**Keywords:** hypogonadotropic hypogonadism, non‐functioning pituitary adenoma, pituitary tumour, quality of life, surgery, transsphenoidal

## Abstract

**Objective:**

The effect of transsphenoidal surgery (TSS) on the hypothalamic‐pituitary‐gonadal (HPG) axis in patients with non‐functioning pituitary adenomas (NFPA) is underexplored, especially in women. Our aim was to investigate the HPG axis function before and after TSS, its association with quality of life (QoL), and to identify factors that predict its recovery after TSS.

**Design:**

This was a prospective cohort study.

**Patients:**

One hundred twenty‐two consecutive patients, 73 men and 49 women, with NFPA planned for TSS.

**Measurements:**

Endocrine function and QoL were assessed before and 12 months after TSS. Psychological well‐being was evaluated using the Psychological General Well‐Being index (PGWBi).

**Results:**

Before surgery, 50 of 73 (68%) men had hypogonadotropic hypogonadism (HH), of whom four (8%) recovered. Eight of 13 (62%) women of reproductive age had preoperative HH, of whom 5 (63%) regained regular menstrual cycles. Preoperative central hypothyroidism (*p* = 0.046) and male sex (*p* = 0.027) were predictors of persistent HH.

The median total PGWBi score increased in both men and women following surgery (*p* < 0.001). Comparing psychological well‐being within subgroups in patients with preoperative HH, categorised by sex (men or women of reproductive age) and postoperative recovery status, only women of reproductive age who recovered their HPG axis experienced improved psychological well‐being.

**Conclusions:**

HH is frequent in men and women with NFPA planned for TSS. Postoperative HPG axis recovery rate is higher in women and is associated with improved QoL.

## 1 Introduction

Hypopituitarism due to non‐functioning pituitary adenomas (NFPA) is common, exceeding 50% among patients who require transsphenoidal surgery (TSS) [1]. Of the hypothalamic‐pituitary hormonal axes, the hypothalamic‐pituitary‐gonadal (HPG) axis is especially vulnerable to mass effect on the pituitary gland [2], resulting in hypogonadotropic hypogonadism (HH). TSS is the primary treatment for NFPA [3]. Still, the influence of TSS on the HPG axis and reproductive health remains largely underexplored [4].

Most studies on HH‐related to pituitary adenomas are made on men, and only a few studies have explored HH before and after TSS. A recent systematic review based on six studies showed that two‐thirds of men with NFPA had HH before TSS, and the recovery rate was 16% [4]. Another systematic review of both men and women with NFPA who underwent TSS reported recovery of the HPG axis in 21% of the patients, and deterioration in 5% [1].

Not much is known about predictors of HPG axis recovery following TSS. However, in a study on patients with prolactinoma, larger tumours and higher baseline prolactin had an impact on recovery after treatment with cabergoline [5]. In another study on men with NFPA, a larger primary tumour/residual tumour volume ratio had a negative impact on HPG axis recovery [2]. Therefore, the need for prospective studies on recovery of the HPG axis was recently highlighted [6], especially in women [7]. It has been shown that HH in women is frequently left untreated, and the prevalence of estrogen substitution is lower than other hormonal substitutions in women with hypopituitarism [8].

The understanding of the relationship between quality of life (QoL) and HPG axis recovery following TSS remains limited. In one study, higher postoperative QoL was associated with testosterone substitution in men [9]. The aim of this prospective study was therefore to investigate HPG axis function and its impact on QoL before and after TSS in patients with NFPA. Furthermore, we aimed to identify factors predicting HPG axis recovery and to investigate sex‐specific disparities related to HH.

## 2 Materials and Methods

### 2.1 Design and Study Population

This was a prospective study, the Gothenburg Pituitary Tumour Study (GoPT), conducted between September 2015 and December 2021, where all patients over 18 years of age, planned for TSS, and who could provide informed consent, were asked to participate (*n* = 228) [10]. Of 228 patients scheduled for TSS, 39 declined participation in the study. Of 189 participants, 60 did not have NFPA, 3 dropped out after surgery, and 4 died within 12 months following surgery. Thus, data from 122 patients operated for NFPA were eligible for analysis.

### 2.2 Patient and Tumour Characteristics

Before surgery, all but two patients underwent magnetic resonance imaging of the sellar region with and without gadolinium‐contrast‐enhanced T1‐ and T2‐weighted sequences (Achieva 3.0 T, Philips Healthcare, Andover, MA, USA). Two patients underwent computed tomography scans prior to surgery. All imaging studies were re‐reviewed by a neuroradiologist, where tumour size was classified dichotomously as micro‐ or macroadenoma (≥1 cm in any dimension), compression of the optic chiasm, and cavernous sinus invasion according to Knosp were evaluated.

### 2.3 Definition of Hypopituitarism

Pituitary function was evaluated pre‐ and post‐operatively by an endocrinologist and later confirmed by another endocrinologist, asserting that every evaluation was consistent with established criteria for hypopituitarism. The pituitary function was evaluated 1 week, 1 month, 6 months, and 12 months after TSS. If the criteria were met at 12 months, or at any of the previous follow‐up visits, and hormone replacement therapy had been initiated, the patient was considered being deficient in that axes.

### 2.4 HH

Male HH was defined as testosterone levels below 10 nmol/L in combination with normal or low LH, and FSH < 12 IU/L. Female HH in postmenopausal women (50 years of age or older) was defined as FSH and LH under < 20 IU/L, and in premenopausal women (under 50 years of age) as estradiol < 30 pmol/L in combination with LH < 15 IH/L and FSH < 10 IU/L, and/or amenorrhea or oligomenorrhea in accordance with previous studies [11]. For comparability, testosterone, LH, and FSH values obtained from external laboratories were adjusted to the upper limit of their reference intervals and normalised to the central laboratory's range.

### 2.5 Adrenal Insufficiency

Adrenal insufficiency was defined as morning cortisol < 100 nmol/L with no ongoing glucocorticoid treatment. Normal levels were defined as > 350 nmol/L. If uncertainty, an intravenous corticotrophin stimulation test was performed according to guidelines [12].

### 2.6 Central Hypothyroidism

Central hypothyroidism was defined as low free thyroxine and with inappropriately non‐elevated TSH, compared to the established reference interwall for the specific laboratory assay [13].

### 2.7 Growth Hormone (GH) Deficiency

GH deficiency was confirmed using a GH stimulation test except in patients with low serum IGF‐1 concentration in combination with ≥ 3 pituitary hormone deficiencies [14]. Confirming stimulation test (insulin tolerance test or arginine + GHRH) was not assessed routinely before surgery, but performed 6−12 months after surgery if GH replacement treatment was considered relevant.

### 2.8 Diabetes Insipidus

Diabetes insipidus was defined as a 24 h urine volume exceeding 2.5 L, accompanied with specific urinary gravity below 1.005 (normal reference range 1.005−1.030) and serum osmolarity over 300 mOsm/kg, or serum sodium over 145 mmol/L, together with treatment response of desmopressin with subsequent symptom relief. If uncertainty, a water‐deprivation test was performed [15].

### 2.9 QoL Assessment

The Psychological General Well‐Being index (PGWBi) questionnaire [16] was used before and 12 months after pituitary surgery to assess psychological well‐being. PGWBi is a 22‐item questionnaire, with six domains (anxiety [five items], depressed mood [three items], positive well‐being [four items], self‐control [three times], general health [three items], and vitality [four items]). Each item has a 6‐point scale, with a total score ranging from 0 to a maximum of 110. A higher score mirrors a better self‐reported psychological well‐being.

The PGWBi questionnaire was chosen as an indicator of QoL since psychological well‐being is closely linked to the function of the HPG axis [17, 18], is validated and considered both reliable, consistent, and sensitive to evaluate psychological well‐being in clinical populations across age and sex strata [18, 19].

### 2.10 Statistical Analysis

Numbers and percentages are used to present categorical data, means with standard deviation (SD) to present normally distributed continuous variables, and median and interquartile range for non‐normally distributed continuous variables.

For comparison between two groups, the Student's *t* test was used for normally distributed data and the Mann–Whitney *U* test for non‐normally distributed data. Fisher's exact test was used for comparisons between categorical variables. For within‐group comparisons of PGWBi scores before and 12‐months after TSS, the Wilcoxon signed‐rank test was used.

To identify predictors of preoperative HH, a uni‐ and multivariable logistic regression model was used and included the following variables: sex, age, body mass index (BMI), adrenal insufficiency, central hypothyroidism, GH deficiency, diabetes insipidus, cavernous sinus invasion, compression of the optic chiasm, and reoperation. To identify factors predicting HPG axis recovery, a comparable logistic regression analysis was used.

To assess the association between QoL and postoperative hormonal deficiencies, multiple linear regression was used. The interaction effect of the variables that showed significance was further tested by creating a variable of interaction.

All analysis were performed with SPSS (v.29.0.2.0 20; IBM Corp., Armonk, NY, USA).

### 2.11 Ethics

The study was conducted in adherence to the approval of the Regional Research Ethics Committee in Gothenburg, Sweden (Reference number: 387‐15), and was in accordance with the guidelines of the World Medical Association Declaration of Helsinki. Prior to entering the study, all participants provided written informed consent.

## 3 Results

### 3.1 Baseline Characteristics

In this study, 122 patients with NFPA were included, 73 (60%) men and 49 (40%) women. The mean ± SD age was 61 ± 14 years, and the mean BMI 28 ± 5 kg/m^2^. All patients except one had a macroadenoma, that is, a tumour larger than 1 cm, 110 (90%) had an optic nerve/chiasmatic compression, and 46 (38%) had cavernous sinus invasion (Knosp ≥ 3). Of 122 patients, 30 (25%) had undergone previous surgery and were scheduled for a reoperation (Table 1). Eighty‐two patients (67%) had at least one pituitary hormonal deficiency: 75 (61%) had HH, 31 (25%) adrenal insufficiency, 45 (37%) central hypothyroidism, 27 (22%) GH deficiency, and 5 (4%) had diabetes insipidus. Of 75 patients with HH before surgery, 50 (67%) were men, and 25 (33%) were women, of whom 13 were of reproductive age (< 50 years of age).

**Table 1:** Characteristics of the study population (*n* = 122).

Men	73 (60)
Women	49 (40)
Age, years	61 ± 14
BMI	28 ± 5
Macroadenoma	121 (99)
Cavernous sinus invasion (Knosp ≥ 3)	46 (38)
Compression of the optic chiasm	110 (90)
Reoperation	30 (25)
Any hormonal deficiency	82 (67)
Hypogonadotropic hypogonadism	75 (61)
Adrenal insufficiency	33 (27)
Central hypothyroidism	46 (38)
Growth hormone deficiency	27 (22)
Diabetes insipidus	5 (4)

*Note:* Presented data as mean ± standard deviation or *n* (%).

Abbreviation: BMI, body mass index.

### 3.2 Predictors of HH Before Surgery

In a univariable analysis, HH before surgery was associated with adrenal insufficiency (*p* < 0.001) and central hypothyroidism (*p* < 0.001; Table 2).

**Table 2:** Predictors of hypogonadotropic hypogonadism before surgery (*n* = 122).

	No HH (*n* = 47)	HH (*n* = 75)	*p*
Sex			0.06
Men	23 (49)	50 (67)	
Women	24 (51)	25 (33)	
Age, years	61 ± 2	61 ± 2	0.524
BMI	26.7 ± 4.25	28.6 ± 5.3	0.057
Compression of the Chiasm	39 (83)	71 (95)	0.057
Cavernous sinus invasion (Knosp ≥ 3)	17 (36)	29 (39)	0.697
Adrenal insufficiency	3 (6)	30 (50)	< 0.001
Central hypothyroidism	7 (15)	39 (52)	< 0.001
Growth hormone deficiency	6 (13)	21 (28)	0.072
Diabetes insipidus	0 (0)	5 (7)	0.155
Prolactin	468 ± 421	564 ± 518	0.501
Reoperation	11 (23)	19 (25)	1.00

Abbreviations: BMI, body mass index; HH, hypogonadotropic hypogonadism.

Variables with *p* values < 0.2 were included in a multivariable binary logistic regression that showed that central hypothyroidism (*p* = 0.008), adrenal insufficiency (*p* = 0.04), compression of the chiasm (*p* = 0.027), and high BMI (*p* = 0.023) were associated with preoperative HH.

### 3.3 HH After Surgery

**Figure 1:**
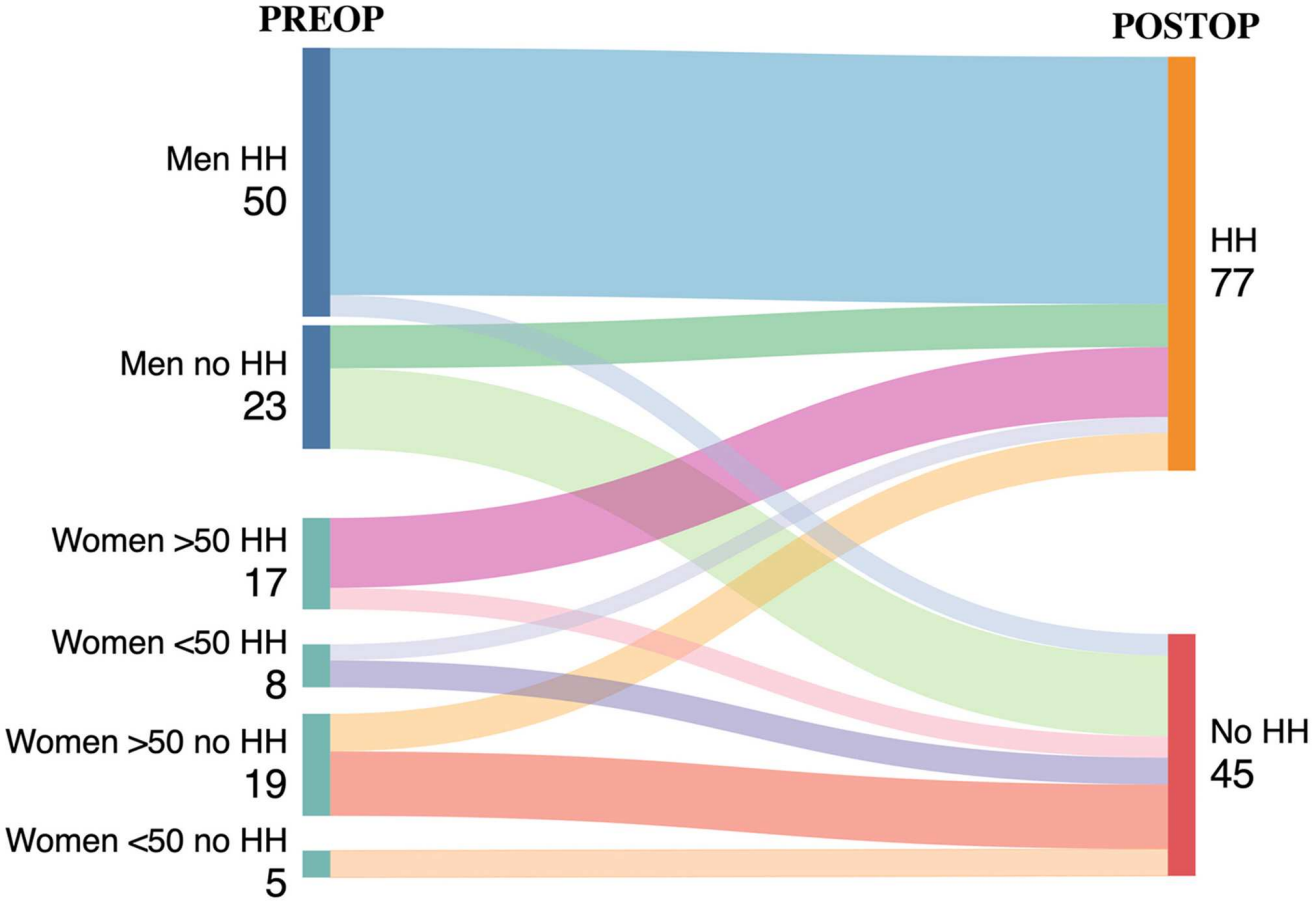
Sankey‐diagram illustrating change in hypothalamic‐pituitary‐gonadal axis function before and 12 months after surgery in men and women. HH, hypogonadotropic hypogonadism; Postop, postoperative; Preop, preoperative.

At the 12‐month follow‐up after surgery, 15 of the 47 (32%) patients (eight of 23 men and seven of 24 women) without preoperative HH had developed a new postoperative HH, and 13 of the 75 (17%) patients with preoperative HH recovered their HPG axis function. Only 4 of 50 (8%) men recovered their HPG axis, compared to 9 of 25 (36%) women. Among the 8 women of reproductive age with preoperative HH, 5 (63%) regained regular menstrual cycles. Altogether, before surgery, 75 (61%) had HH, and after surgery, 77 (63%) (Figure 1).

### 3.4 Predictors for Recovery of the HPG Axis

In a univariable analysis of predictors of HPG axis recovery 12 months after surgery, central hypothyroidism (*p* < 0.001), reoperation (*p* = 0.030), GH deficiency (*p* = 0.015), and male sex (*p* = 0.007) were associated with less likelihood of recovering the HPG axis (Table 3).

**Table 3:** Predictors for recovery of the hypothalamic‐pituitary‐gonadal axis (*n* = 75).

	No recovery (*n* = 62)	Recovery (*n* = 13)	*p*
Sex			0.007
Men	46 (92)	4 (8)	
Women	16 (64)	9 (36)	
Age, years	63 ± 14	53 ± 19	0.069
BMI	28.7 ± 5.2	28.1 ± 5.8	0.418
Compression of the Chiasm	58 (94)	13 (100)	1.00
Cavernous sinus invasion (Knosp ≥ 3)	26 (42)	3 (23)	0.133
Adrenal insufficiency	28 (45)	2 (15)	0.063
Central hypothyroidism	38 (61)	1 (7)	< 0.001
Growth hormone deficiency	21 (34)	0 (0)	0.015
Diabetes insipidus	5 (8)	0 (0)	0.580
Prolactin	527 ± 496	699 ± 595	0.219
Reoperation	19 (31)	0 (0)	0.030
HH diagnoses > 6 months ago	39 (63)	6 (46)	0.353

*Note:* Presented data as mean ± standard deviation or *n* (%).

Abbreviation: HH, hypogonadotropic hypogonadism.

Variables with *p* values < 0.2 (except of cavernous sinus invasion because of several missing cases) were included in a multivariable binary logistic regression that showed that central hypothyroidism (*p* = 0.046) and male sex (*p* = 0.027) were associated with persistent HH.

### 3.5 Sex Disparities of HPG Axis Recovery

A subgroup univariable analysis of possible predictors of HPG axis recovery 12 months after surgery for men and women, respectively, showed a positive association with preoperative high testosterone levels in men (*p* = 0.047) and a negative association for central hypothyroidism and HPG axis recovery for women (*p* = 0.003; Table 4).

**Table 4:** Baseline clinical and biochemical characteristics stratified by surgical outcome and sex.

A. Men	No Recovery (*n* = 46)	Recovery (*n* = 4)	*p*
Age, years	64 ± 13	61 ± 22	0.924
BMI	29.0 ± 3.9	27.5 ± 2.7	0.474
Compression of the Chiasm	43 (93)	4 (100)	1.00
Cavernous sinus invasion (Knosp ≥ 3)	23 (50)	1 (25)	0.336
Adrenal insufficiency	21 (46)	2 (50)	1.00
Central hypothyroidism	28 (61)	1 (25)	0.348
Growth hormone deficiency	17 (37)	0 (0)	0.285
Diabetes insipidus	2 (4)	0 (0)	1.00
LH	1.58 ± 0.99	2.85 ± 2.94	0.555
FSH	3.79 ± 3.99	4.92 ± 4.46	0.620
Testosterone	2.12 ± 1.98	4.33 ± 2.36	0.047
Prolactin	405 ± 360	345 ± 88	0.903
Reoperation	16 835)	0 (0)	0.292
HH diagnoses > 6 months ago	28 (61)	3 (75)	1.00

B. Women	No Recovery (*n* = 16)	Recovery (*n* = 9)	*p*
Age, years	61 ± 18	50 ± 18	0.095
BMI	28.1 ± 7.9	28.4 ± 9.3	0.934
Compression of the Chiasm	15 (94)	9 (100)	1.00
Cavernous sinus invasion (Knosp ≥ 3)	3 (19)	2 (22)	1.000
Adrenal insufficiency	6 (38)	0 (0)	0.057
Central hypothyroidism	10 (63)	0 (0)	0.003
Growth hormone deficiency	4 (25)	0 (0)	0.260
Diabetes insipidus	3 (19)	0 (0)	0.280
Prolactin	788 ± 645	856 ± 661	0.815
LH	2.18 ± 2.67	2.92 ± 1.52	0.123
FSH	6.09 ± 5.93	9.02 ± 5.36	0.138
Reoperation	3 (19)	0 (0)	0.280
HH diagnoses > 6 months ago	11 (69)	3 (33)	0.115

*Note:* Data is presented as mean ± standard deviation or *n* (%).

Abbreviations: BMI, body mass index; HH, hypogonadotropic hypogonadism.

### 3.6 QoL Before and After Surgery

**Figure 2:**
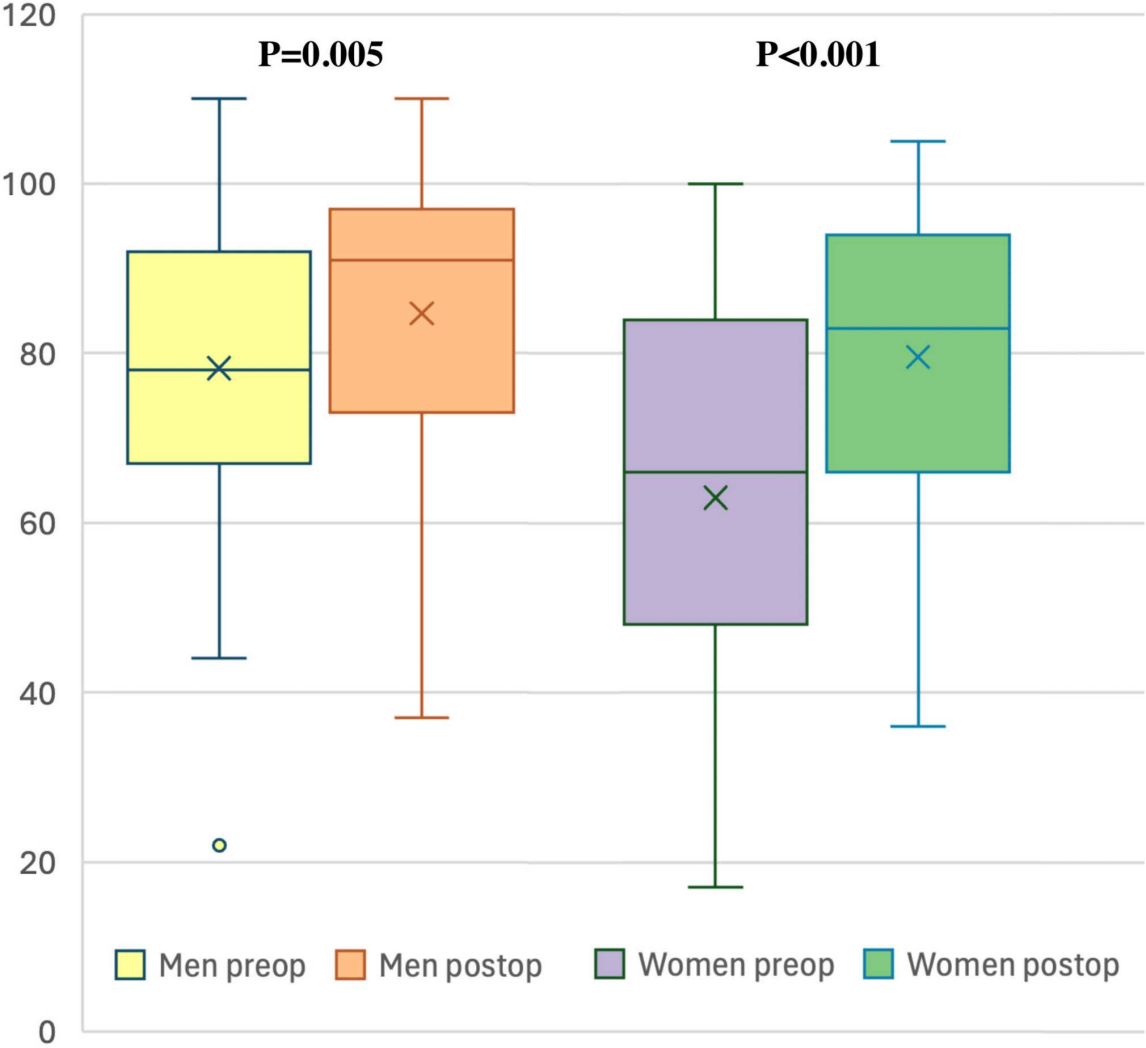
Median PGWBi total score in men and women before and after transsphenoidal surgery. Higher scores indicate higher psychological well‐being. Postop, postoperative, PGWBi, The Psychological General Well‐Being Index; Preop, preoperative.

The median total PGWBi score in the whole cohort improved from 77 (IQR 58−90) before surgery to 90 (IQR 71−97) 12 months after surgery (*p* < 0.001). In men, the median total PGWBi score improved from 78 (IQR 67−92) before surgery to 91 (IQR 73−97), 12 months after surgery (*p* = 0.005). In women the median total PGWBi score improved from 66 (IQR 48‐84) before surgery to 83 (IQR 66−94), 12 months after surgery (*p* < 0.001; Figure 2).

The difference in median improvement was higher in women (17 points) than in men (13 points; *p* = 0.002).

For the 62 patients with HH who did not recover HPG axis function, the median total PGWBi score improved from 78 (IQR 64−92) before surgery to 90 (IQR 68−97), 12 months after surgery (*p* = 0.005).

For the 13 patients who recovered their HPG axis the PGWBi score improved from 74 (IQR 41−88) before surgery to 93 (IQR 81−98) 12 months after surgery (*p* = 0.004; Table 5).

**Table 5:** PGWBi before and after surgery in men and women stratified by HPG axis recovery status.

	PGWBi before surgery (median, IQR)	PGWBi after surgery (median, IQR)	*p*
All patients (*n* = 122)	77 (IQR 58‐90)	90 (IQR 71‐97)	< 0.001
All men (*n* = 73)	78 (IQR 67‐92)	91 (IQR 73‐97)	0.005
All women (*n* = 49)	66 (IQR 48‐84)	83 (IQR 66‐94)	< 0.001
Preop HH who did not recover (*n* = 62)	78 (IQR 64‐92)	90 (IQR 68‐97)	0.005
Preop HH who did recover (*n* = 13)	70 (IQR 41‐88)	93 (IQR 81‐98)	0.004
Women < 50 years who did recover (*n* = 5)	24 (IQR 20‐77)	94 (IQR 76‐99)	0.043
Women < 50 years who did not recover (*n* = 3)	66 (IQR 48‐68)	76 (IQR 68‐83)	0.109
Men who did recover (*n* = 4)	71 (IQR 58‐95)	90 (IQR 77‐92)	0.144
Men who did not recovery (*n* = 46)	82 (IQR 74‐94)	91 (IQR 75‐98	0.120

*Note:* Presented data as median (Interquartile range ([QR]), with PGWBi score ranging from 0 to 110.

Abbreviations: HH, hypogonadotropic hypogonadism; HPG axis, hypothalamic‐pituitary‐gonadal axis; PGWBi, The Psychological General Well‐Being index; Postop, postoperative; Preop, preoperative.

The difference in median improvement was higher in those who did recover their HPG axis (19 points) compared to those who did not (12 points; *p* = 0.009).

### 3.7 QoL and Other Hormonal Deficiencies

Multiple linear regression showed that recovery of the HPG axis was associated with improved QoL, with a 13‐point increase in the PGWBi score (*p* = 0.016). Postoperative adrenal insufficiency was associated with reduced QoL, with a 10‐point decrease (*p* = 0.018). The interaction term between recovery of the HPG axis and adrenal insufficiency was not significant (*p* = 0.303), suggesting that the effect of recovery of the HPG axis on QoL is independent of adrenal insufficiency. Other pituitary disorders (central hypothyroidism, GH deficiency, and diabetes insipidus) had no significant effect on improvement in QoL.

### 3.8 QoL—Subgroup Analysis of Men and Women of Reproductive Age by HPG Axis Recovery

Five women of reproductive age recovered their HPG axis, and their PGWBi score improved from 24 (IQR 20−77) before surgery to 94 (IQR 76−99) 12 months after surgery (*p* = 0.043). For the three women of reproductive age who did not recover their HPG axis, the PGWBi score was 66 (IQR 48−68) before surgery and 76 (IQR 68−83) 12 months after surgery (*p* = 0.109).

**Figure 3:**
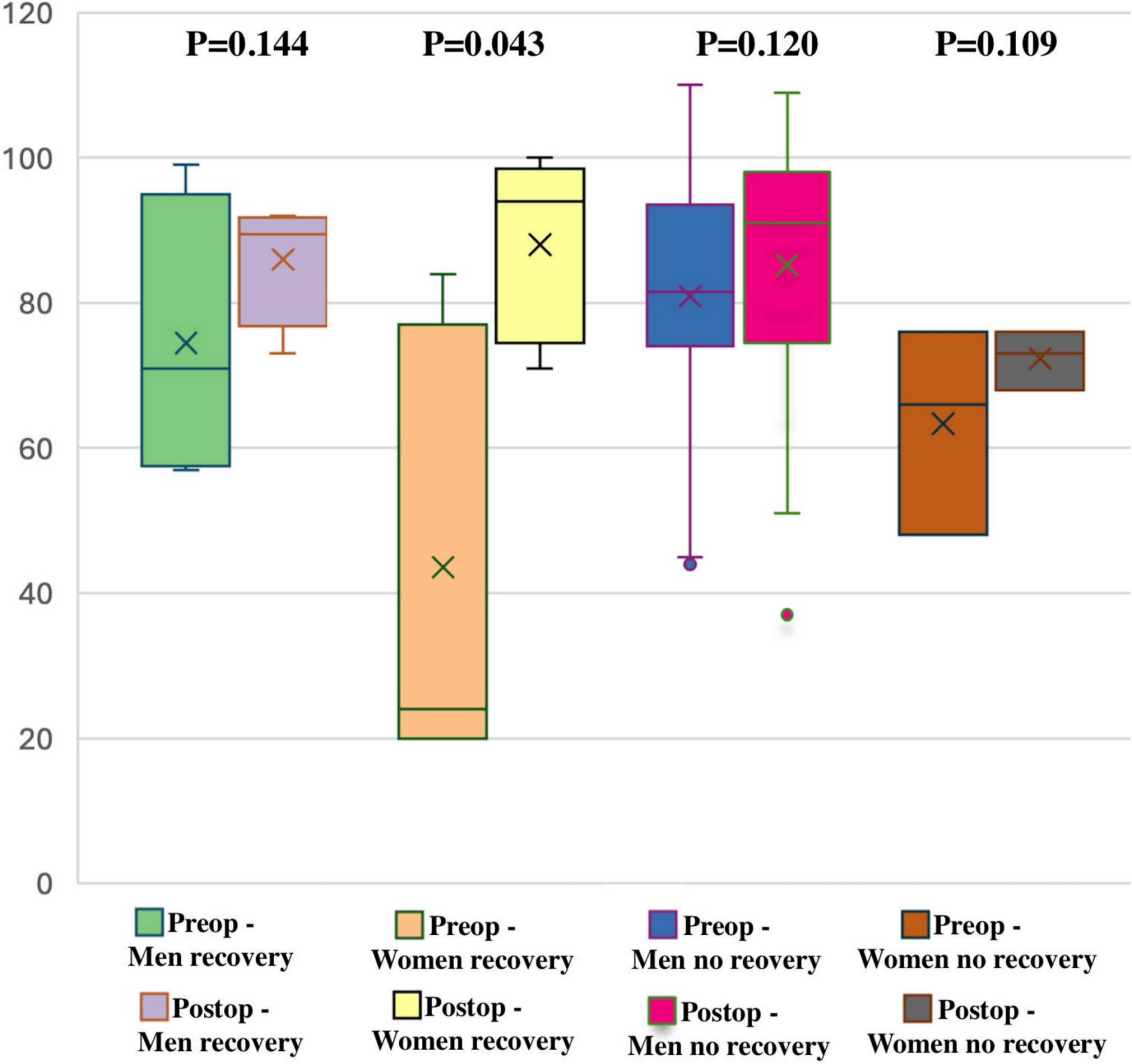
Median PGWBi total score in men and women before and after transsphenoidal surgery who did or did not recover their hypothalamic‐pituitary‐gonadal axis. Median PGWBi total score in men (*n* = 50, 4 recovered) and women of reproductive age (*n* = 8, 5 recovered) with preoperative hypogonadotropic hypogonadism before and after transsphenoidal surgery, with comparisons before and after surgery and difference between groups by sex and HPG axis recovery status. Higher scores indicate higher psychological well‐being. PGWBi, The Psychological General Well‐Being Index; Postop, postoperative; Preop, preoperative.

Among the 46 men who did not recover their HPG axis, the PGWBi score was 82 (IQR 74−94) before surgery and 91 (IQR 75−98) 12 months after surgery (*p* = 0.120). For the four men who did recover their HPG axis, the PGWBi was 71 (IQR 58‐95) before surgery and 90 (IQR 77−92), 12 months after surgery (*p* = 0.144; Table 5, Figure 3). Given the small sample size for this subgroup analysis, these results should be interpreted with caution.

### 3.9 Change in the PGWBi's Sub‐Domains by Postoperative HPG Axis Function and Sex

**Figure 4:**
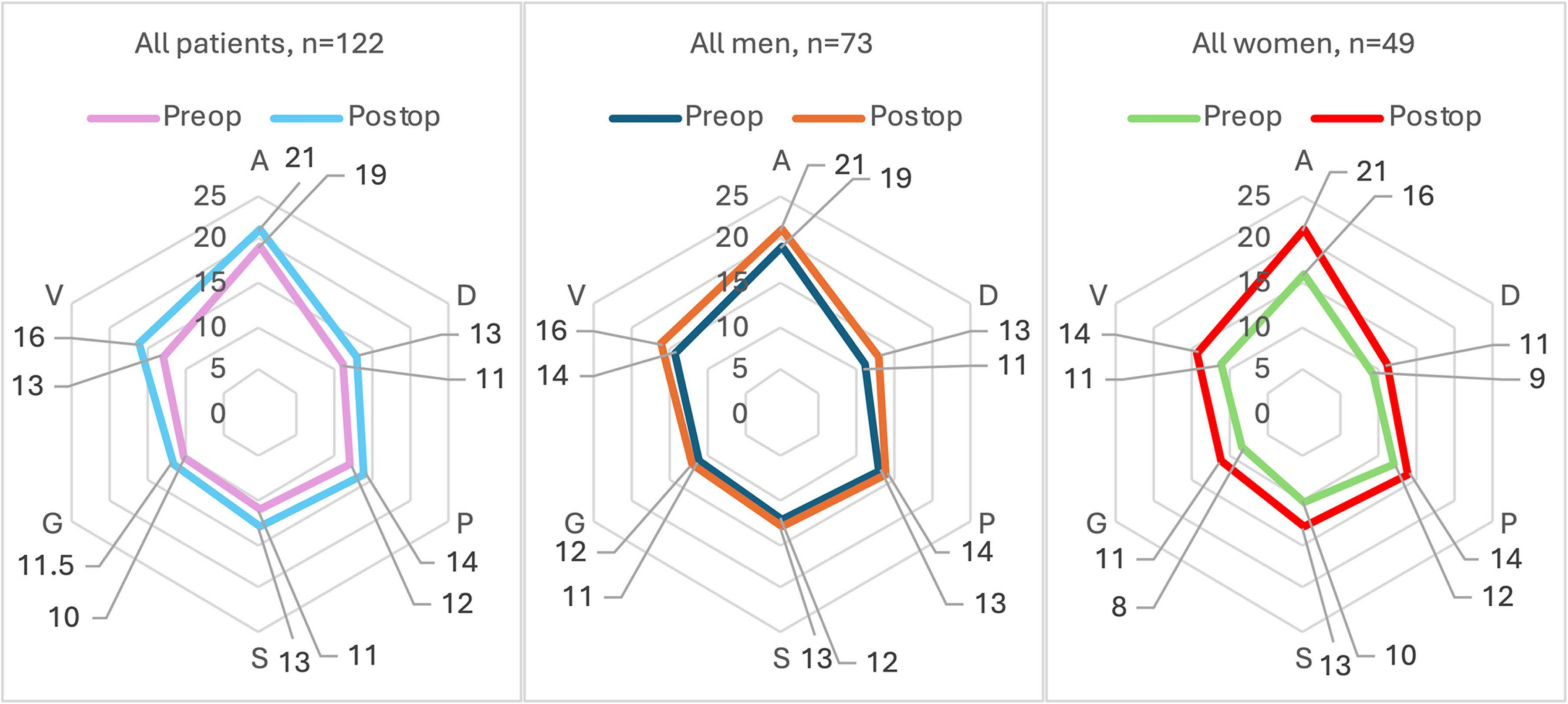
Spider web diagram of each sub‐domain from the PGWBi before and after surgery first for the entire cohort, then for all men and for all women. Higher scores indicate higher psychological well‐being. Abbreviations and score range A, anxiety (0−25); D, depressed mood (0−15); G, general health (0−15); P, positive well‐being (0−20); PGWBi, The Psychological General Well‐Being index; Postop, postoperative; Preop, preoperative; S, self‐control (0−15); V, vitality (0−20).

In women (*n* = 49), all six PGWBi's domains improved (*p* < 0.001). In men (*n* = 73) all domains except general health (*p* = 0.081) improved; anxiety (*p* = 0.002), depressed mood (*p* = 0.023), positive well‐being (*p* = 0.007), self‐control (*p* = 0.002), and vitality (*p* = 0.014), (Figure 4).

**Figure 5:**
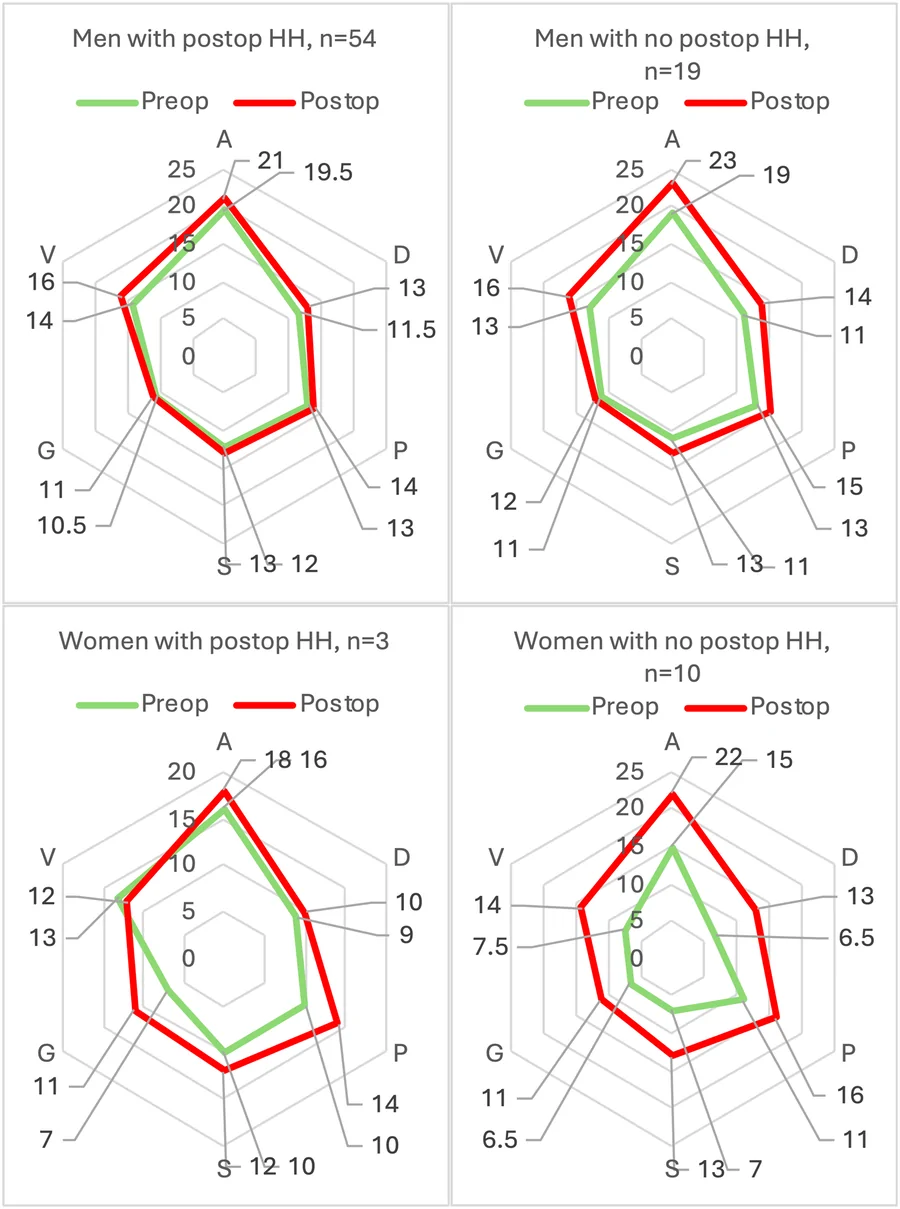
Spider web diagram of each sub‐domain from the PGWBi before and after surgery for all men and for all women of fertile age by postoperative hypothalamic‐pituitary‐gonadal axis status. Higher scores indicate higher psychological well‐being. Abbreviations and score range A, anxiety (0−25); D, depressed mood (0−15); G, general health (0−15); P, positive well‐being (0−20); PGWBi, The Psychological General Well‐Being index; Postop, postoperative; Preop, preoperative; S, self‐control (0−15); V, vitality (0−20).

The subgroup of women in reproductive age who either developed HH post‐operatively or did not recover their HPG axis (*n* = 3), did not report any improvement in any domain, whereas women of reproductive age who did not develop HH or recovered their HPG axis (*n* = 10) reported higher scores in all domains; anxiety (*p* = 0.012), depressed mood (*p* = 0.011), positive well‐being (*p* = 0.017), self‐control (*p* = 0.012), general health (*p* = 0.021), and vitality (*p* = 0.020). In men who either developed HH or did not recover their HPG axis (*n* = 54) no improvement was seen in any domain, while men who did not develop HH or recovered their HPG axis (*n* = 19) an improvement in all domains was observed: anxiety (*p* = 0.005), depressed mood (*p* = 0.010), positive well‐being (*p* = 0.034), self‐control (*p* = 0.002), general health (*p* = 0.039), and vitality (*p* = 0.024; Figure 5).

## 4 Discussion

This study shows that HH is common in both men and women with NFPA planned for TSS. The HPG axis recovery rate after TSS was higher in women than in men, the function of the HPG axis had an impact on psychological well‐being 12 months after pituitary surgery, and HPG axis recovery was associated with a larger improvement of QoL.

Women reported worse psychological well‐being than men before and after surgery, however their improvement was greater. Psychological well‐being improved in the total cohort, regardless of HPG axis status, but the improvement was more marked in those who recovered their HPG axis. These sex differences have also been observed in previous studies with women with chronic health condition report worse QoL compared to men [20]. Reduced psychological well‐being has been associated with the symptom profile present in menopausal women [21].

In a subgroup analysis, only women of reproductive age who recovered their HPG axis showed improved psychological well‐being. When analysing the different domains of the PGWBi, men and women of reproductive age with postoperative intact HPG axis improved in all domains, while those with postoperative HH did not improve in any domains.

Even though women and patients who recovered their HPG axis showed a significantly greater improvement in PGWBi total score, psychological well‐being improved in the entire cohort. This improvement was observed independently of other anterior pituitary functions and regardless of HPG axis recovery status. This could indicate that the hormonal replacement therapy is effective and well‐tolerated. In a previous study, males with HH testosterone replacement therapy even scored higher in psychological well‐being than those with preserved HPG axis, suggesting that testosterone therapy *per se* may have a positive impact on the patients' wellbeing [9].

The proportion of patients who developed HH was in line with previous studies [1], and was comparable between men and women, although none of the five women of reproductive age developed HH.

In this study, higher testosterone levels in men and preserved TSH production in women were predictors of HPG axis recovery. Besides male sex, preoperative central hypothyroidism, and reoperation were predictors for persistent HH. The total recovery rate was in line with previous studies [1, 4].

A relatively high preoperative testosterone level may indicate a less severe HPG axis dysfunction with a greater chance of recovery. Since the HPG axis is one of the most sensitive axes to pituitary mass effect [2], preoperative adrenal insufficiency and central hypothyroidism for women indicate a more general impact on the pituitary gland, resulting in a more severe form of hypopituitarism with a lower chance of recovery. This is supported by a previous study where women with hypopituitarism had lower sex hormone levels if at the same time having adrenal insufficiency [22].

Previous studies have reported increased risk of HH with larger tumour size and high prolactin levels [2, 5]. Although exact tumour volume was not measured in this study, parameters indicating large tumour size, such as sinus cavernous invasion, compression of the optic chiasm, or high prolactin, were not associated with a lower recovery rate of HH, however compression of the optic chiasm was associated with preoperative HH. Additionally, this study observed an association between preoperative HH and adrenal insufficiency and high BMI.

Comparing gonadal function in men and women presents several challenges. Women after menopause may be of physiological interest, but not necessarily clinically relevant in this context. Excluding postmenopausal women may introduce age‐related bias, whereas excluding older men would result in small comparison groups. The definition of HH and choice of cut‐offs are also important; more stringent cut‐offs may fail to capture recovery of gonadal function, while overly liberal cut‐offs may overestimate the prevalence of HH. Our definition and cut‐offs were prespecified and consistent with previous studies [11]. Given the physiological variability in estradiol levels, resumption of menstrual cycles was considered the most reliable indicator of gonadal recovery in premenopausal women.

The difference in recovery of the HPG axis between women and men may be explained by the menstrual cycle's sensitivity to pituitary tumour impact, leading women to seek medical care early due to oligo‐amenorrhea. In contrast, men present with more vague symptoms and subsequently experience longer diagnostic delay. This is supported in earlier studies where women are diagnosed with non‐functioning pituitary microadenomas at a younger age than men [23]. At the same time, it could be argued that tumors in postmenopausal women may have been present for a longer period due to the absence of HH‐related symptoms. Earlier diagnosis in women may reduce the severity of the mass effect from the pituitary tumour on the HPG axis, thereby increasing the likelihood of recovery. Another possibility is that women and men could have different thresholds for clinically relevant HPG axis recovery. A partial recovery of the HPG axis could be enough for women to regain their ovarian cycle, while it might not be sufficient for men to regain spermatogenesis and libido. The postoperative management could also play a role, where a common approach is to wait several months to ascertain that they regain their menstrual cycle in women, while in men, the approach is more reliant on laboratory tests. The patient, as well as the physician, might opt to initiate replacement of testosterone early instead of giving the HPG axis a chance to recover after pituitary surgery.

A strength of this study is the prospective design to investigate HH and QoL before and after TSS in patients with NFPA, with a specific focus on comparing outcomes in men and women. By investigating sex disparities, this study provides new information on women's reproductive health, a subject previously addressed as a knowledge gap [7]. One limitation of this study was that GH deficiency before surgery was not assessed as a routine on all patients, which may result in an underestimation of its true prevalence and a possible predictor of HPG axis recovery. Given the few women of fertile age with NFPA and the few men who recover their HPG axis, further studies with larger cohorts are needed to ensure broader applicability.

In the meantime, this study provides important information that can be incorporated into the preoperative consultation with the patient, with the possibility of a more tailored person‐centred postoperative follow‐up. In women planning pregnancy, the expectation of HPG axis recovery could even in borderline cases be a factor that pivots the decision to proceed with surgery. For men, a more cautious approach directed towards monitoring testosterone and the possible need for testosterone replacement therapy is reasonable.

In conclusion, women are more likely than men to recover their HPG axis after TSS. Higher preoperative testosterone in men and preserved TSH production after surgery in women are predictors of HPG axis recovery after TSS. The QoL improves after surgery, and a preserved or recovered HPG axis contributes to this improvement independently of the function of the other axes. This study underscores that a personalised approach in the treatment decision‐making process is important for patients with NFPA planned for TSS. The result brings hope for women in fertile age with NFPA with preoperative HH who want to start a family.

## Author Contributions

The authors confirm that the manuscript is original and has not been submitted elsewhere. All authors have made a substantial contribution to the work and approved the final version of the manuscript.

## Conflicts of Interest

G.J. has served as a consultant for Novo Nordisk and AstraZeneca and received lecture fees from Novo Nordisk, Pfizer, and Pharmanovia. None of these activities was related to the work presented in this paper. The other authors declare no conflicts of interest.

## Data Availability

Some or all datasets generated during and/or analysed during the current study are not publicly available but are available from the corresponding author on reasonable request: Victor Hantelius, MD. Department of Medicine, Sahlgrenska University Hospital, Gothenburg, Sweden, and Department of Internal Medicine and Clinical Nutrition, Institute of Medicine, Sahlgrenska Academy, University of Gothenburg, Gothenburg, Sweden E‐mail: victor.hantelius@vgregion.se.
